# Therapeutic benefits of niraparib tosylate as radio sensitizer in esophageal squamous cell carcinoma: an in vivo and in vitro preclinical study

**DOI:** 10.1007/s12094-022-02818-7

**Published:** 2022-04-01

**Authors:** Yuzhong Cui, Wei Huang, Feng Du, Xiaoyang Yin, Lei Feng, Baosheng Li

**Affiliations:** 1 Tianjin Medical University Cancer Institute and Hospital, Tianjin, 300060 China; 2 National Clinical Research Center for Cancer, Tianjin, 300060 China; 3 Key Laboratory of Cancer Prevention and Therapy, Tianjin, 300060 China; 4 Tianjin’s Clinical Research Center for Cancer, Tianjin, 300060 China; 5 Department of Oncology, Zibo Municipal Hospital, Zibo, 255400 China; 6 Shandong First Medical University and Shandong Academy of Medical Sciences, Jinan, 250117 China; 7 Department of Radiation Oncology, Shandong Cancer Hospital and Institute, Shandong First Medical University and Shandong Academy of Medical Sciences, 440 Jiyan Road, Huaiyin District, Jinan, 250117 Shandong China

**Keywords:** Esophageal cancer, In vivo, Niraparib tosylate, Preclinical, Radiosensitivity

## Abstract

**Purpose:**

Esophageal squamous cell carcinoma is associated with high morbidity and mortality rate for which radiotherapy is the main treatment modality. Niraparib, a Poly (ADP-ribose) polymerase 1 inhibitors (PARPi) was previously reported to confer radiosensitivity in different malignancies including non-small cell lung cancer. In this study, we assessed the in vivo ability of niraparib in conferring radiosensitivity to esophageal squamous cell carcinoma cells.

**Materials and methods:**

In this study, KYSE-30 and KYSE-150 cell lines were selected as in vivo esophageal squamous cell carcinoma models. The experimental groups were: niraparib tosylate alone, radiotherapy alone, control (no intervention), and combination therapy (radiotherapy + niraparib tosylate). Cell cytotoxicity assay, colony formation assay, flow cytometry, immunofluorescence, Western blotting, immunohistochemistry, lentivirus transfection analysis, and xenograft models were used for confirming radiosensitizing ability of niraparib and to investigate the possible cellular mechanism involved in radiosensitization.

**Results:**

The colony formation efficiency of the combination group was significantly much lower than that of the single radiation group (*P* < 0.01). Cell cytotoxicity assay demonstrated a significant reduction in proliferation of irradiated cells after treatment with niraparib tosylate compared to niraparib tosylate alone (*P* < 0.01). Cell apoptosis significantly increased in the combination group compared to either niraparib tosylate or radiotherapy alone (*P* < 0.01). Rate of tumor suppression rate was significantly high in the combined treatment group (*P* < 0.01) but, significantly decreased in nude mice. Western blot and lentivirus infection model suggested overexpression of FANCG genes to confer radiosensitivity.

**Conclusion:**

These results suggest that the synergistic effect of niraparib tosylate and radiation may be related to the down-regulation of FANCG.

**Supplementary Information:**

The online version contains supplementary material available at 10.1007/s12094-022-02818-7.

## Introduction

Esophageal cancer (EsC) is currently the eighth prevalent type of cancer and ranks 6th as the leading cause of mortality, with a 5-year survival rate ranging from 15 to 25% [1]. It is estimated that a global increase of 35% in the incidence of EsC may occur between 2018 and 2030 [2]. Among the different histological subtypes of EsC, squamous cell carcinoma (SCC) and esophageal adenocarcinoma (EAC) accounts for more than 95% of all EsCs [3]. Among the different treatment modalities, radiotherapy plays a pivotal role in the management of EsC as revealed by the landmark clinical trial comparing the benefits of chemoradiotherapy after surgery with surgery alone [9].

Despite the perceived benefits of chemoradiotherapy, a subset of patients still experienced disease progression or recurrence mainly due to pre-existing radiotherapy resistant clones [4]. Pre-existing radiotherapy resistant clones may had led to disease progression which could have been due to upregulation of certain metabolic pathways that potentiates survival of malignant cells despite the accumulation of double-strand breaks (DSBs) caused by radiotherapy [5]. Among the different genes involved with radiotherapy resistance, the FANCG genes were observed to be up-regulated in the radioresistant PC-3 cell line [6]. Till now, 22 genes of the FA pathway have been identified which play an important role in DNA repair [7]. The breast cancer susceptibility genes, BRCA1 and BRCA2, are also known to be involved in the FA pathway. The FA-BRCA pathway is involved in the repair of DNA damage and maintaining genome stability, and is closely related to the occurrence, development, prognosis, and drug resistance of some tumors [8]. Loss of FA gene leads to the development of head and neck, esophageal, gastrointestinal, vulvar, and anal cancers [8]. Mutation in FA genes triggers cancer, especially acute myeloid leukemia and squamous cell carcinoma [9].

The PARP inhibitors (PARPi) are known to stall the DNA repair in tumor cells, resulting in the accumulation of DSBs leading to genomic instability and cell death [10]. Niraparib is a highly selective PARPi, used frequently in ovarian epithelial cancer, fallopian tube cancer, and other diseases. However, whether it has radiotherapy sensitization effect on esophageal squamous cell carcinoma and the potential mechanism of action remain to be further clarified. In this preclinical study, KYSE-30 and KYSE-150 cell lines were used to understand the mechanism of radiosensitivity of esophageal SCC treated with niraparib. This study aims to provide new ideas for the treatment of esophageal SCC and theoretical support for the clinical application of PARPi in combination with radiotherapy.

## Methodology

### Selection of cell line and cell culture

The proliferation of five human esophageal squamous cell carcinoma cells KYSE-30, KYSE-150, KYSE-450, KYSE-510, and TE-1 were detected by CCK8 assay. The cell line with the lowest and highest IC_50_ value for niraparib and radiation sensitivity were selected for subsequent experiment. The human esophageal squamous cell carcinoma cell (ESCC) line KYSE-30 and KYSE-150 were purchased from Procell Life Technology Co., Ltd. (Wuhan, China), and the cells were cultured in DMEM culture medium (Thermo Fisher Scientific, USA) supplemented with 10% fetal bovine serum (Hyclone, USA) and 1% penicillin/streptomycin (Thermo Fisher, USA) at 37 °C in an atmosphere of 95% air and 5% CO_2_.

### Cytotoxicity assay

To confirm the radiation sensitization effect of niraparib tosylate, CCK8 was used to detect the effect of different treatments at different time points [11]. Niraparib tosylate was obtained from the Zai Lab (Shanghai) Co., Ltd, China. The purity of compounds was more than 99%. KYSE-30 and KYSE-150 cells were cultured in 96-well plates at 3000 cells/well for 24 h. These were divided into four groups; blank control, radiation (4 Gy) treatment, niraparib tosylate (5 µmol/l) treatment and niraparib tosylate and radiation combined treatment groups (combination treatment). Following the treatment, 10 µl enhanced cell counting Kit-8 solution (CCK8, BIOSS Technologies, Inc., Beijing, China) was added to each well at different time points. Then, the absorbance at 450 nm for each well was measured and the cell survival rate was calculated (cell survival rate (%) = (value of experimental group—blank group value)/ (value of control group—blank group value). Each experiment was repeated thrice independently.

### Colony formation assay

Cells were seeded in 60 × 60 mm cell culture plates at 1000 cells/plate, and were cultured for 24 h to allow the cells adhere to the wall. KYSE-30 and KYSE-150 cells were seeded and divided into four groups, as mentioned above. First, the cells were incubated for 8–10 days. Later, the colonies were fixed with 4% paraformaldehyde for 15 min and stained with 1% crystal violet solution for 15 min at room temperature. Subsequently, the colonies consisting ≥ 50 cells were counted under a microscope [12]. The colony formation rate (%) was calculated as follows: number of colonies counted/number of cells seeded × 100%. The surviving fraction (SF) was determined as follows: SF = number of colonies/(number of cells seeded under the same condition × colony formation rate) [13].

### Flow cytometry analysis of apoptosis

KYSE-30 and KYSE-150 cells were plated in 6-well plates and treated with or without 5 µmol/l niraparib tosylate for 6 h, and then exposed to X-rays (4 Gy). After 24 h, cells were trypsinized and washed with phosphate-buffered saline (PBS). The cell suspensions were stained using the Annexin V-FITC Apoptosis detection kit (BD Biosciences, Beijing, China), as per the manufacturer's protocol, followed by flow cytometric analysis. The apoptosis rate was analysed using FlowJo 7.6 software. All experiments were repeated thrice independently [14].

### Immunofluorescence

The cells were incubated on coverslips in 35 mm dishes and treated with 5 μmol/l niraparib tosylate and radiation at a dose of 4 Gy. Immunofluorescence staining of γ-H2AX (#7631), Bcl-2 (#3498), and Bax (#5023) was performed. The cells were then fixed (4% paraformaldehyde), permeabilized (0.1% Triton X-100), blocked (5% BSA), and then incubated with primary antibodies overnight at 4℃. Subsequently, the cells were incubated with Alexa Fluor pre-adsorbed secondary antibody for 1 h at room temperature (Abcam, ab150117). Nuclei were stained with DAPI (Solarbio, Beijing, China). Thereafter, the single-stained and merged images were acquired using laser confocal microscopy and Zen 3.0 imaging software [15].

### Quantitative real-time reverse transcription PCR

Total RNA was extracted using Trizol (Invitrogen). RNA concentration was measured using the Qubit^®^ RNA Assay Kit in Qubit^®^ 2.0 Fluorometer (Life Technologies, CA, USA) and the purity was determined using the NanoPhotometer^®^ spectrophotometer (IMPLEN, CA, USA). RNA degradation and contamination were assessed on 1% agarose gels. cDNA was synthesized with the Takara-RR047A, and the PCR amplification mixture was 10 μl in total, containing 1 μl cDNA [16]. Primer pairs used were as follows: β-actin: forward 5′-GGCACCACACCTTCTACAATG-3′, reverse 5′-GTGGTGGTGAAGCTGTAG-3′; Bcl-2: forward 5′-GGGAGGATTGTGGCCTTCTT-3′, reverse 5′-GGGCCAAACTGAGCAGAGTC-3′; Bax: forward 5′-CGGGGAGCAGCCCAGAG-3′, reverse 5′-TGAGACACTCGCTCAGCTTC-3′; Caspase-3: forward 5′-ATTTGGAACCAAAGATCATACATGG-3′, reverse 5′-CTGAGGTTTGCTGCATCGAC-3′. Every assay was performed in triplicate and the mRNA levels of β-actin were used as internal standards.

### Western blotting

Western blotting was conducted to determine the effects of niraparib tosylate treatment on the expression of apoptosis-related genes and DNA double-strand breaks. Western blot was used to detect the expression of Bcl-2, Bax, and γ-H2AX in each group [11]. Experimental groupings and cell inoculation were as described above. Cells were collected 24 h after irradiation. The protein was extracted from cells and quantified using a BCA kit (catalog no. P0010s; Beyotime Institute of Biotechnology). A total of 30 µg protein was loaded per lane for western blot analysis. 10% SDS–PAGE and 5% stacking gels were prepared, and the protein samples were electrophoresed. The proteins were then transferred to a polyvinyl difluoride membrane and blocked with 5% BSA or 5% skim milk for 2 h at room temperature. The membranes were then incubated with the following primary antibodies: rH2AX (#7631), Anti-BAX (#5023), Anti-BCL-2 (#3498), Caspase-3 (#9664) (1:1000; Cell Signaling Technology, Inc.), FANCG (10215-1-AP) (1:500; Protein tech Group, Inc.) at 4 °C overnight.β-ACTIN (#4970) (1:1000; Cell Signaling Technology, Inc.) was used as an internal control. Subsequently, the membranes were incubated with anti-rabbit IgG secondary antibody (1:1000; Cell Signaling Technology, Inc) for 1 h at room temperature. Visualization was performed with enhanced chemiluminescence reagent. Image J 1.8.0 processing software was used for gray-scale analysis. Each experiment was repeated thrice independently.

### Xenograft models

Radiosensitization of niraparib tosylate in vivo was evaluated [11]. For this a tumor-bearing nude mouse model of esophageal squamous cell carcinoma KYSE-30 cells was established. Right subcutaneous flank injections were performed on twenty 4–5-week-old BALB/c-SCID female mice (obtained from Beijing Charles River Laboratory Animal Technology Co., Ltd.) with 2 × 10^6^ KYSE-30 cells resuspended in 100 μl PBS. When the tumors reached approximately 70 mm^3^, mice were randomly divided into four groups as follows: Control group (oral PBS), niraparib tosylate group, Radiation group and combined group (oral niraparib tosylate + irradiation). Mice in the treatment groups were anesthetized and subjected to local irradiation (12 Gy) of the tumors. Niraparib tosylate (50 mg/kg) was administered orally once daily for 10 days. Tumor volumes and body weights were measured every 3 days. Tumor volume was calculated as: 1/2 × length × width^2^. Tumor inhibition rate = (average volume of control group—average volume of experimental group)/average volume of control group × 100%. All procedures were performed in a certified biosafety cabinet and carried out in accordance with protocols approved by the Committee for the Care and Use of Laboratory Animals of our institute.

### HE staining and immunohistochemistry

To further observe the morphological structure and growth of tumor in each group, HE staining, Caspase-3 and FANCG immunohistochemical staining were used to detect tumor in each group [17, 18]. The mice were sacrificed at the end of the experiment and the tumor tissues were fixed in 10% formalin, paraffinized, and cut into 5 μm thick sections.

- HE staining.

The slides were dewaxed and stained with hematoxylin–eosin for histological assessments.

(b) Immunohistochemistry.

After microwave pre-treatment in a citrate buffer (pH 6.0; for antigen retrieval), the slides were immersed in 3% hydrogen peroxide for 20 min to block endogenous peroxidase activity. After intensive washing with PBS, the slides were incubated with Caspase-3, FANCG antibodies and then incubated with HRP-conjugated antibodies. The slides were visualized with a DAB Horseradish Peroxidase Color Development Kit (Beyotime, China), and counterstained with hematoxylin. The images were analyzed by Image-Pro Plus 6.0 software.

### Lentivirus transfection

Four groups of tumor-bearing nude mouse transplantation models were subjected to transcriptome high-throughput sequencing, 20 genes with significant expression differences were selected, relevant articles were queried through PubMed and related pathways were queried through KEGG database for analysis, so as to determine the modification and research of FANCG gene. Analyzed lentiviruses with overexpression of FANCG and negative control lentiviruses (Shanghai GeneChem Co., LTD.) were manufactured according to the instructions. In brief, cell suspensions (50,000 cells/ml, 2 ml/well) of KYSE-30 cells were inoculated into six-well plates for 24 h in DMEM with 10% FBS. Then, DMEM without FBS was replaced, lentivirus solution (20 μl 1 × 108 TU/ml) was added for another 16-h incubation and DMEM with 10% FBS was added back for another 72 h. The screening and purification of infected cells was performed by culture in a medium containing puromycin.

### Statistical analysis

All data were presented as the mean ± standard deviation (SD) and were analysed with statistical software SPSS 21.0 (SPSS Inc). Comparisons of continuous variables between the groups were performed using the student’s *t* test. The criterion for statistical significance was taken at *P* < 0.05.

### Survival analysis

The Cancer Genome Atlas (TCGA) data were queried for esophageal squamous cell carcinoma. The Kaplan–Meier survival plot was generated to assess the association between FANCG expression on esophageal squamous cell carcinoma and overall survival [19].

## Results

### Cell culture and cytotoxicity assay

**Fig. 1:**
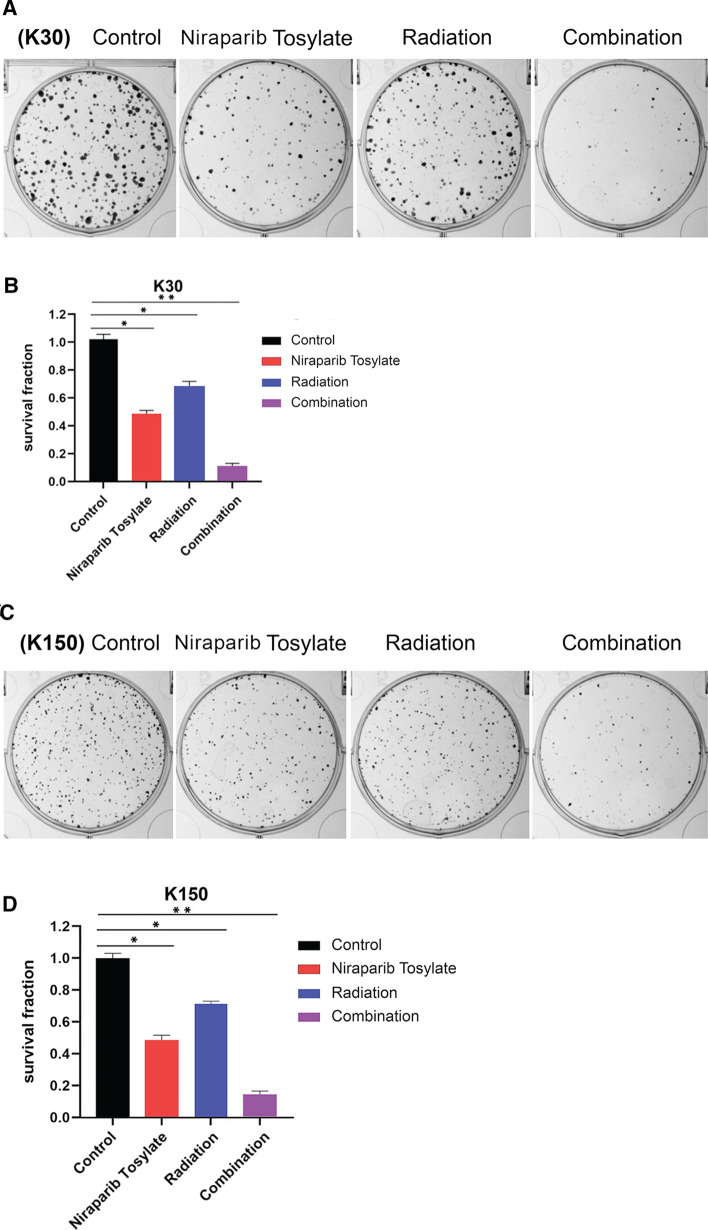
Colony formation assay of four groups. **A**, **B** Formation of colonies for KYSE-30 cell line. **C**, **D** Formation of colonies for KYSE-150 cell line

**Fig. 2:**
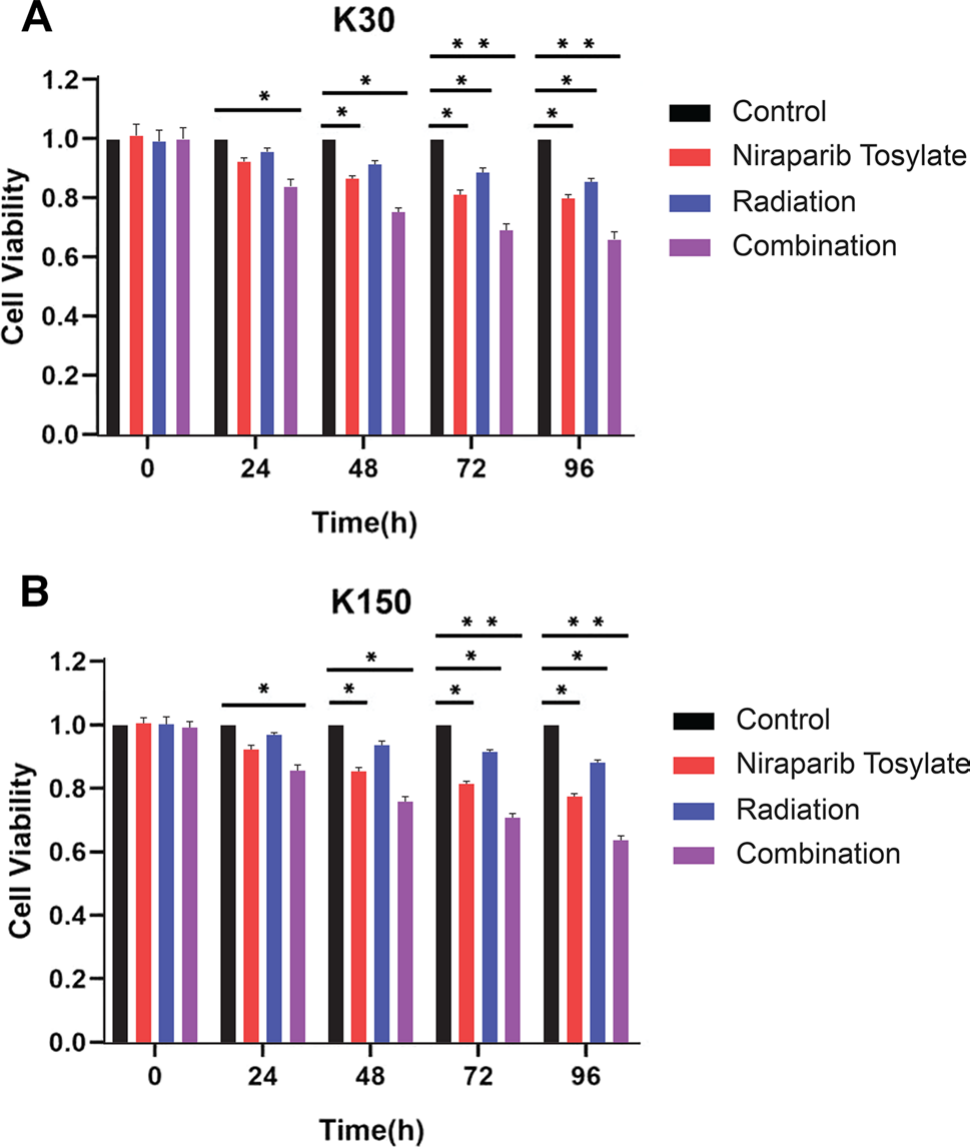
Cytotoxicity assay (CCK8). **A** Cell survival rate for KYSE-30 cell line. **B** Cell survival rate for KYSE-150 cell line

The median inhibitory concentrations (IC50) of niraparib tosylate at 24 h for KYSE-30, KYSE-150, KYSE-450, KYSE-510, and TE-1, respectively, were 39.44 μmol/l, 36.87 μmol, 38.98 μmol/l, 36.69 μmol/l, 19.05 μmol/l (supplementary Fig. 1). TE-1 was highly sensitive to drugs, and the difference was statistically significant compared with the other four (*P* < 0.01). KYSE-30 had the highest radiation sensitivity, while KYSE-150 had the lowest sensitivity, and the difference was not statistically significant (*P* > 0.05). Therefore, two cell lines KYSE-30 and KYSE-150 were selected for the study (supplementary Fig. 2). The radiotherapy dose was 4 Gy, and niraparib tosylate was 5 μmol/l. The colony formation efficiency of combined group was much lower than that of single radiation group (*P* < 0.01) (Fig. 1). CCK8 assay confirmed that the addition of niraparib tosylate significantly reduced the proliferation of irradiated cells (Fig. 2). Therefore, both colony formation assay and CCK8 assay confirmed that niraparib tosylate could improve radiotherapy sensitivity by inhibiting cell proliferation.

### Flow cytometry

**Fig. 3:**
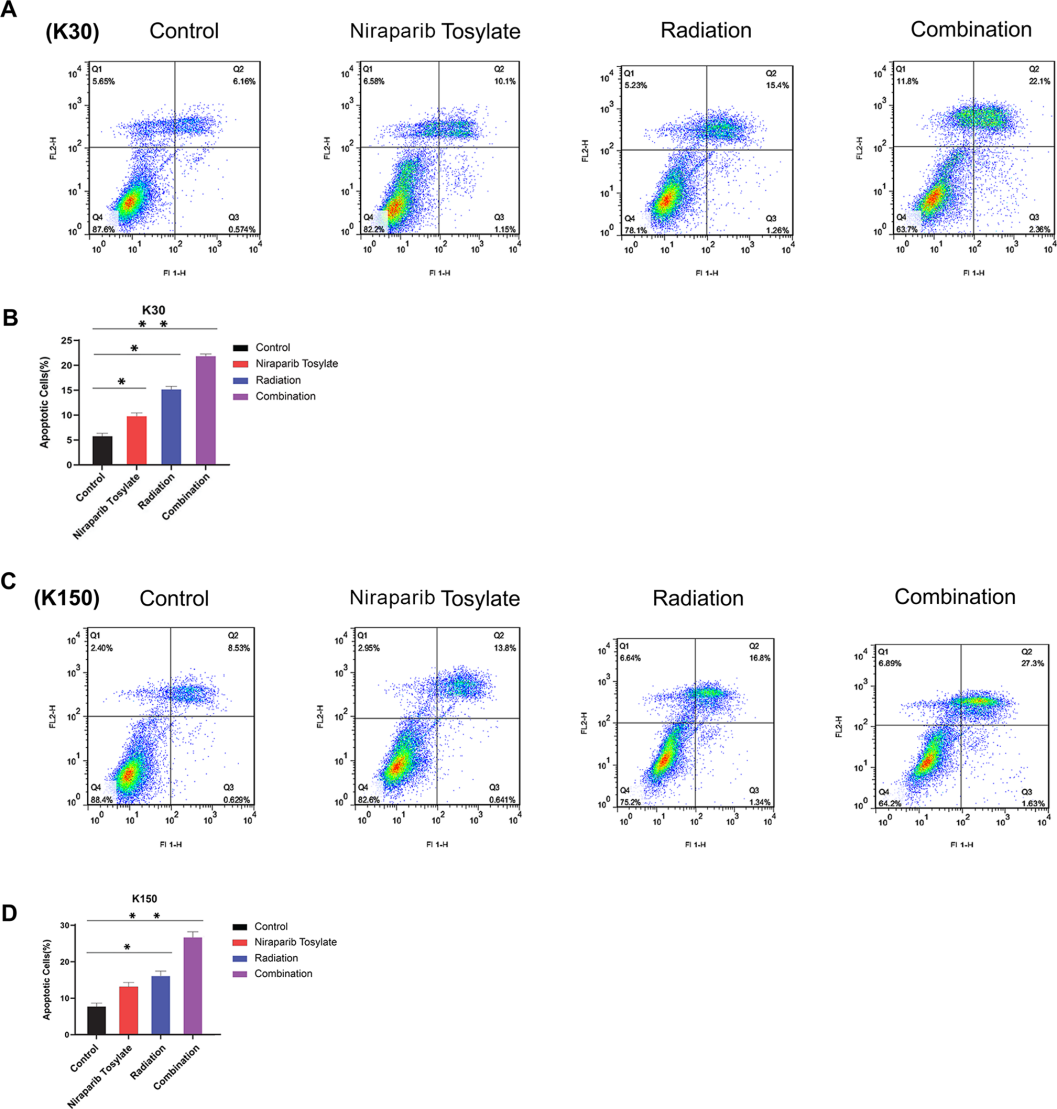
The late cell apoptosis of four groups. **A**, **B** The late cell apoptosis for KYSE-30 cell line. **C**, **D** The late cell apoptosis for KYSE-150 cell line

Compared with the control group, although the late cell apoptosis was steadily increased in the niraparib tosylate and radiotherapy groups (*P* < 0.05), the increase in cell apoptosis was more significant in the combination group (*P* < 0.01) (Fig. 3).

### PCR, immunofluorescence and Western blotting

**Fig. 4:**
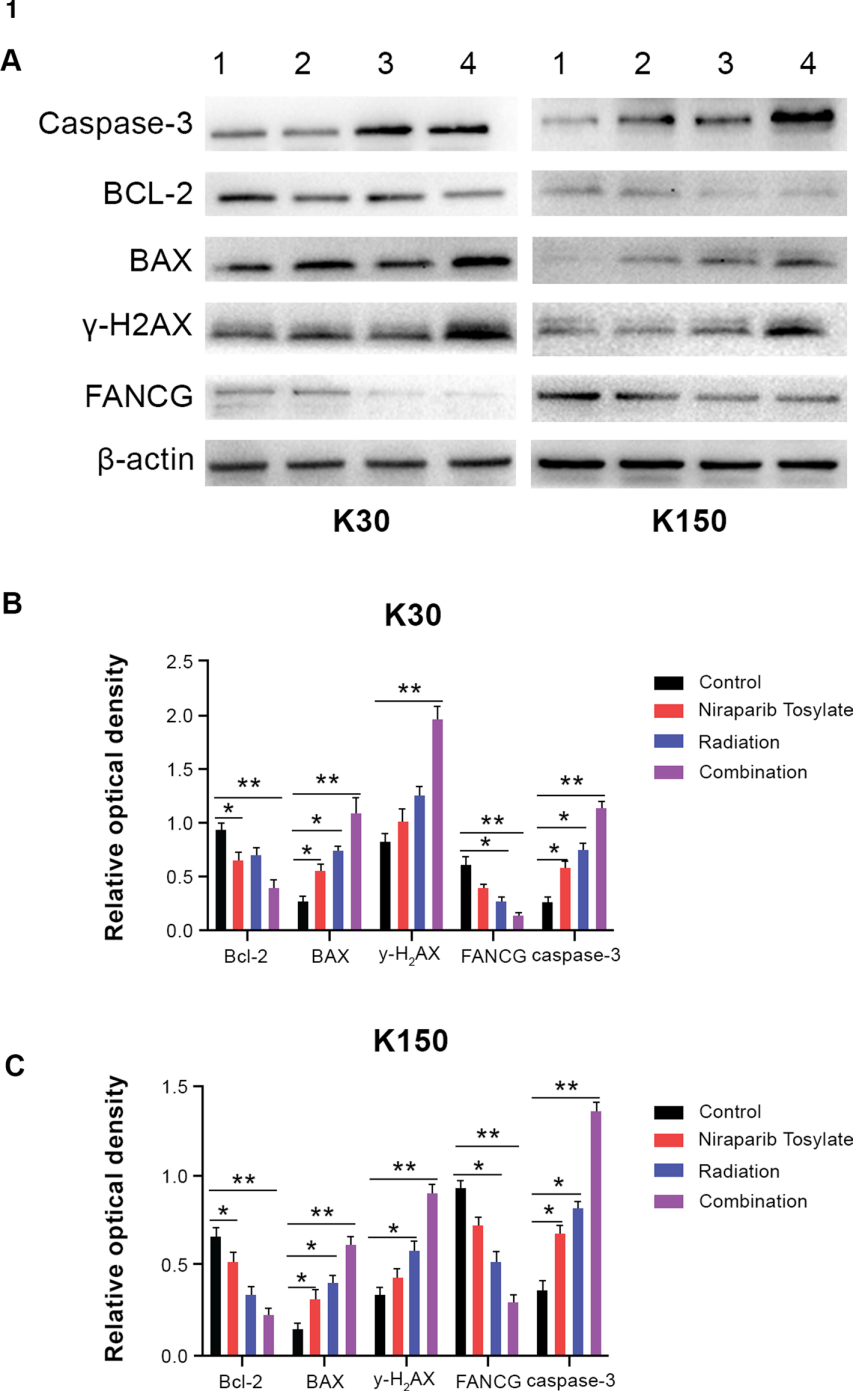

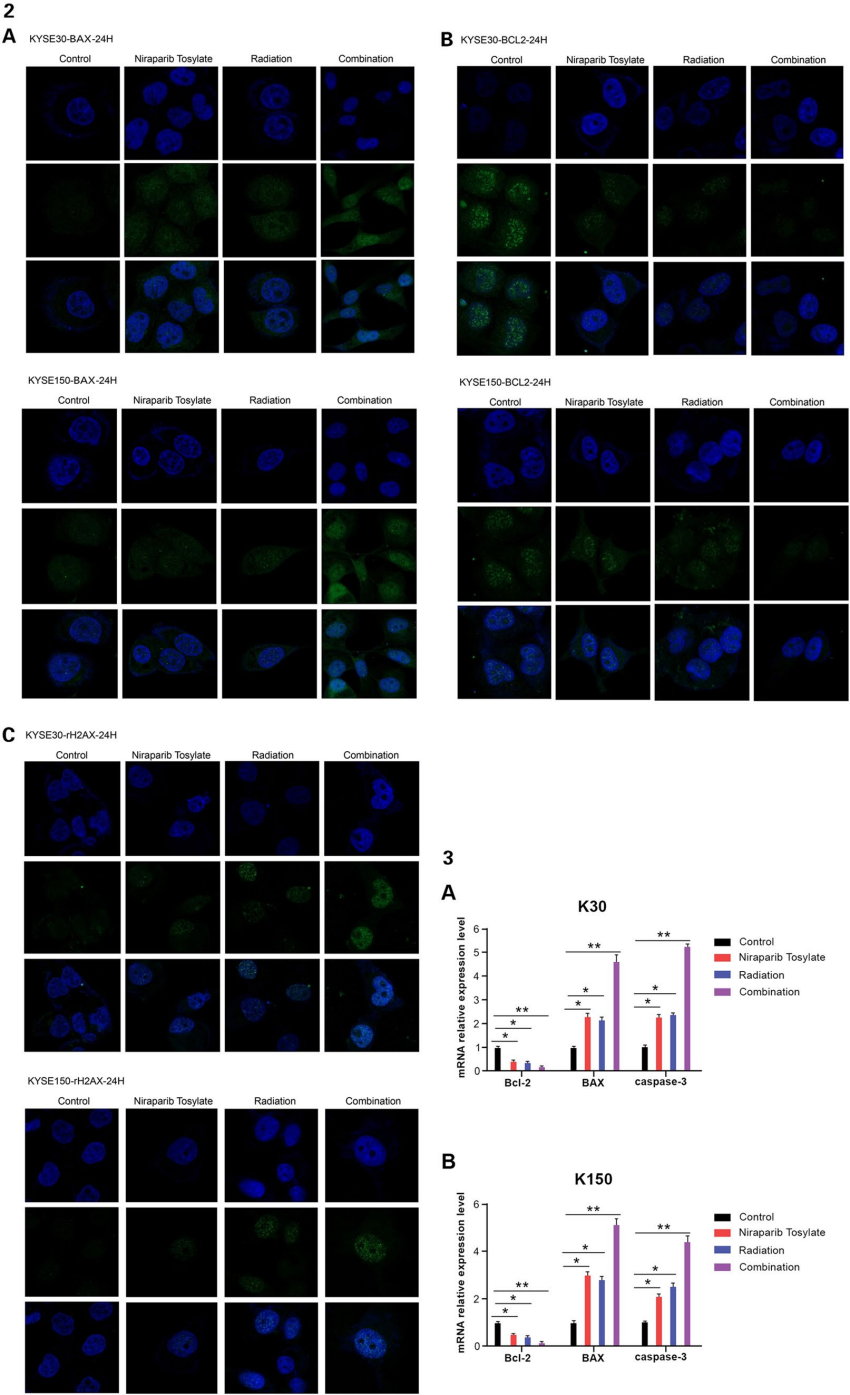
(1) Western blotting of four groups **A** Western blotting for KYSE-30 and KYSE-150 cell lines. **B** Relative optical density for KYSE-30 cell line. **C** Relative optical density for KYSE-150 cell line. (Band 1: control group; Band 2: Niraparib Tosylate group; Band 3: radiation group; Band 4: combination group.) *P < 0.05, **P < 0.01. (2) **A** Immunofluorescence of γH2AX. **B** Immunofluorescence of BAX. **C** Immunofluorescence of BCL-2. (3) mRNA relative expression level among the four groups for **A** KYSE-30 cell line. **B** KYSE-150 cell line. *P < 0.05, **P < 0.01

It was observed that the combination of niraparib tosylate and radiation inhibited the expression of Bcl-2 (*P* < 0.01), up-regulated the expression of Bax and Caspase-3 (*P* < 0.01), and significantly increased the expression of γ-H2AX (*P* < 0.01). Compared with the radiation group, the addition of niraparib tosylate significantly increased the DNA DSBs of apoptotic cells. These results suggest that the role of niraparib tosylate in radiation-induced apoptosis is to promote apoptosis by regulating apoptosis-related genes (Fig. 4-1, -2, -3).

### Xenograft model

**Fig. 5:**
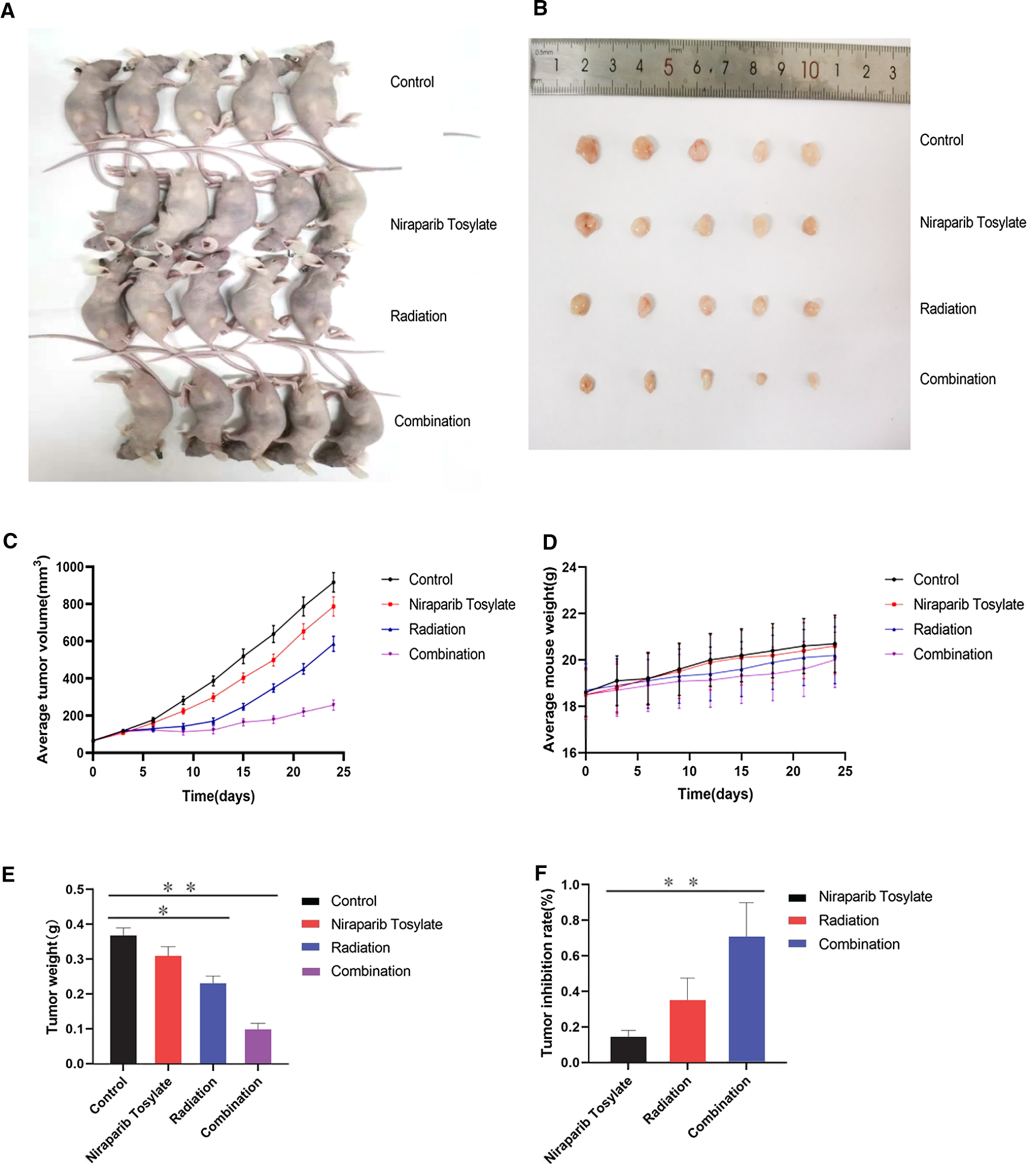
Xenograft model pre-FANCG genetic modification. **A** Four groups of nude mice. **B** Four groups of tumor tissue. **C** Mean tumor volume in the four groups. **D** Average weight of the rats in the four groups. **E** Differences in tumor weight among the four groups. **F** Tumor inhibition rates in three groups

The results showed that in the xenograft tumor model before FANCG gene modification, niraparib tosylate alone had a weak inhibition effect on tumor growth (*P* > 0.05), while both radiotherapy and combination therapy could effectively inhibit tumor growth in the xenograft model (*P* < 0.05), and the combination group had a stronger inhibitory effect (*P* < 0.01). There was no significant difference in body weight among the four groups (*P* > 0.05). These results suggest that niraparib tosylate and radiation have a significant synergistic effect on KYSE-30 xenotransplantation and have little effect on quality of life in nude mice (Fig. 5).

### HE and immunohistochemical staining

HE staining showed increased tumor necrosis in all treatment groups, especially in the combination group, compared with the control group. Caspase-3 staining showed that the expression of Caspase-3 was significantly increased in the combined treatment group, indicating that tumor cell apoptosis was more significant after combined treatment. This reconfirmed the tumor suppressive effect of niraparib tosylate combined with radiotherapy at cellular and animal levels (Supplementary Fig. 3).

### FANCG expression

**Fig. 6:**
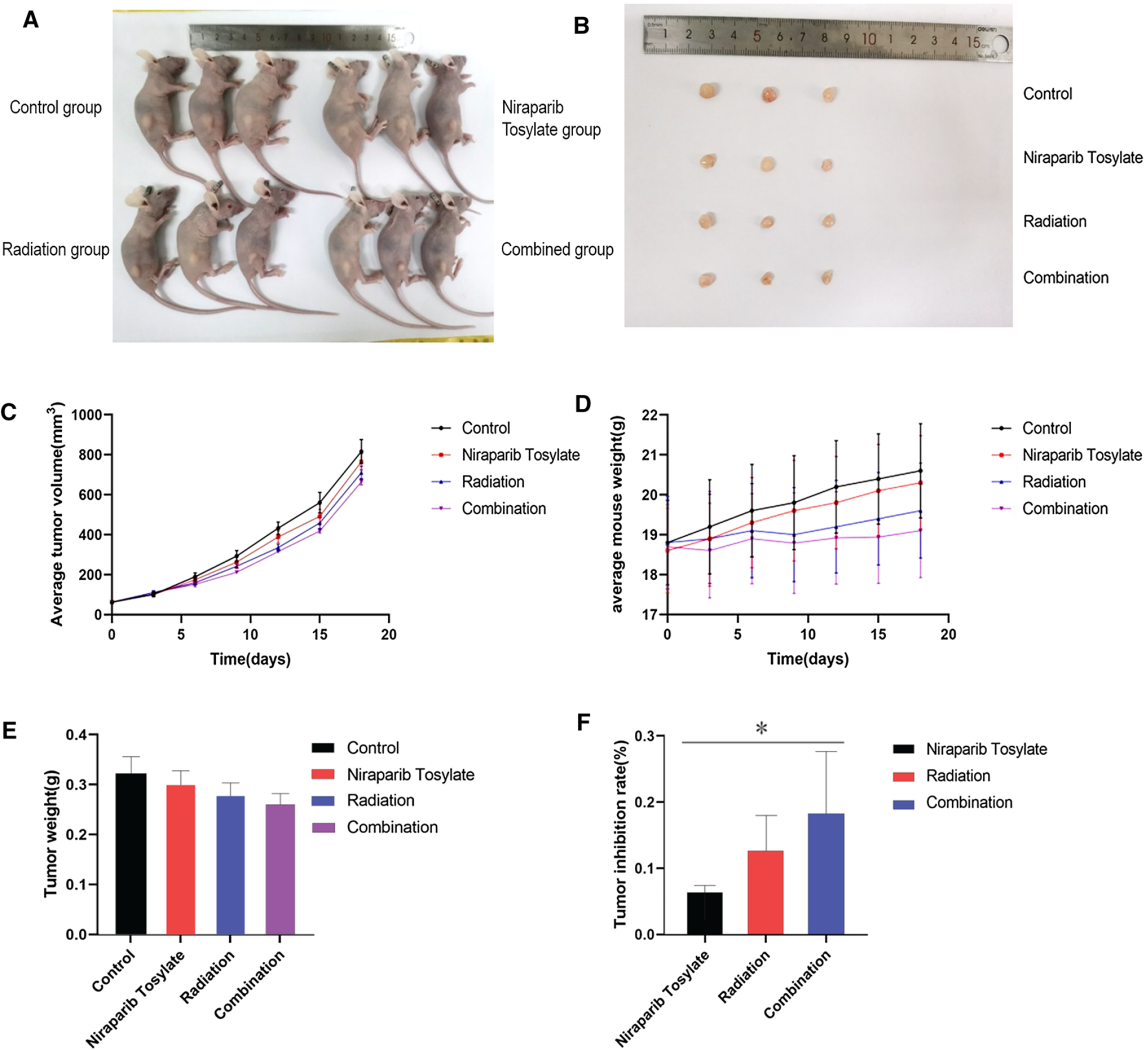
Xenograft model after over expression of FANCG gene. **A** Four groups of nude mice. **B** Four groups of tumor tissue. **C** Mean tumor volume in the four groups. **D** Average weight of the rats in the four groups. **E** Differences in tumor weight among the four groups. **F** Tumor inhibition rates in three groups. (*represents < 0.05, **represents < 0.01)

**Fig. 7:**
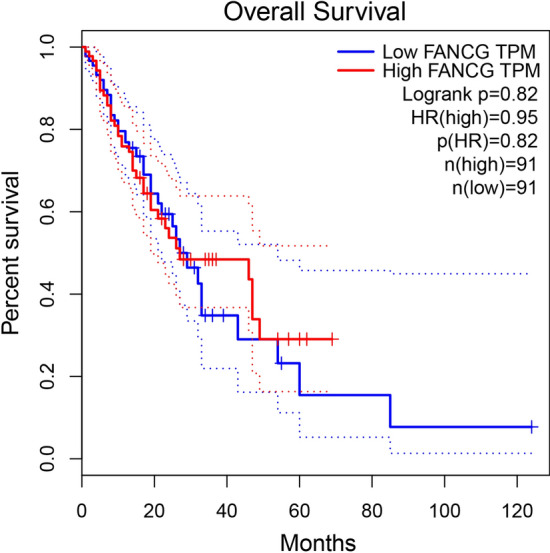
Overall survival analysis of FANCG in esophageal squamous cell carcinoma (based on TCGA data)

**Fig. 8:**
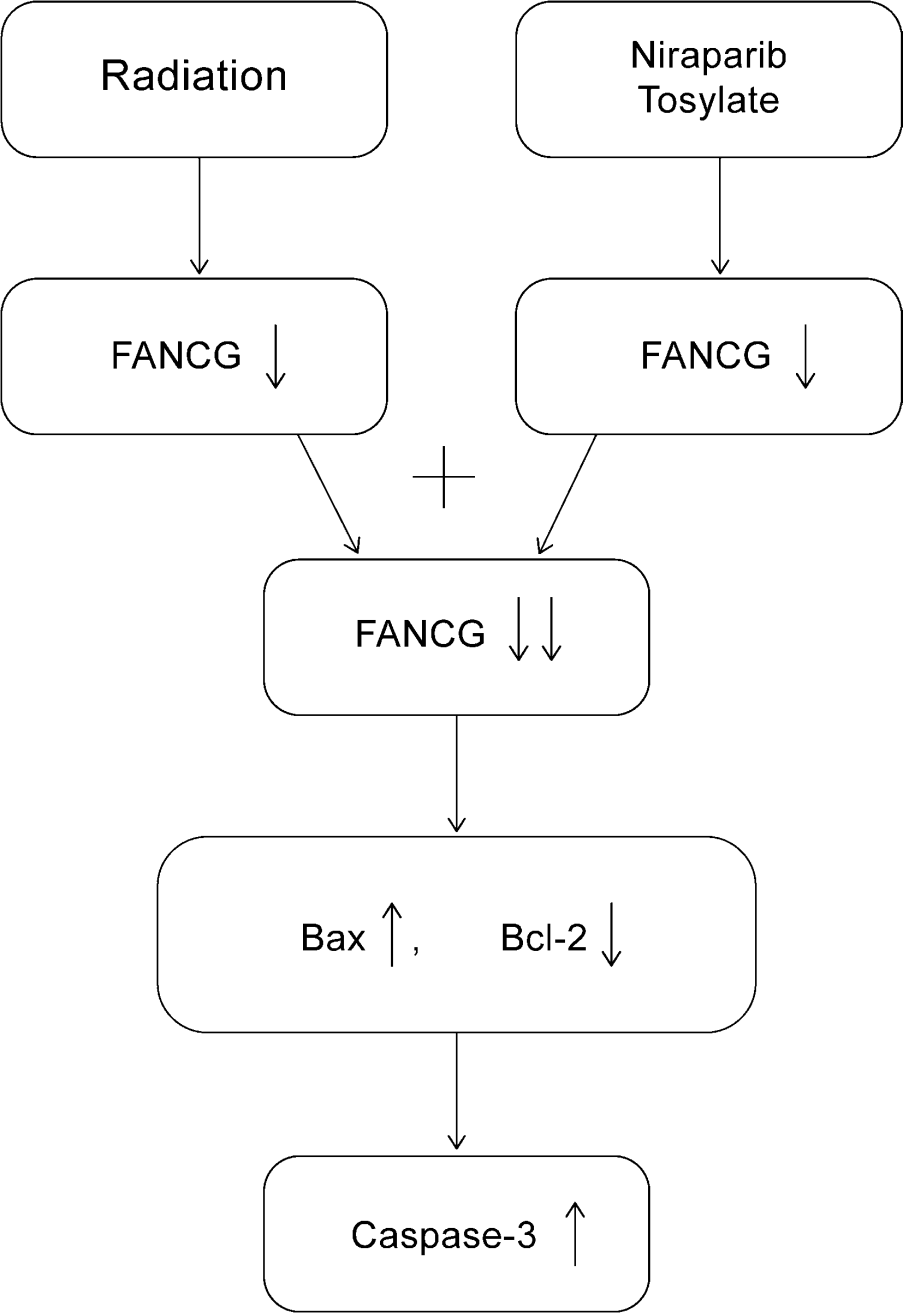
Graphical representation of proposed pharmacological mechanism of Niraparib Tosylate

**Fig. 9:**
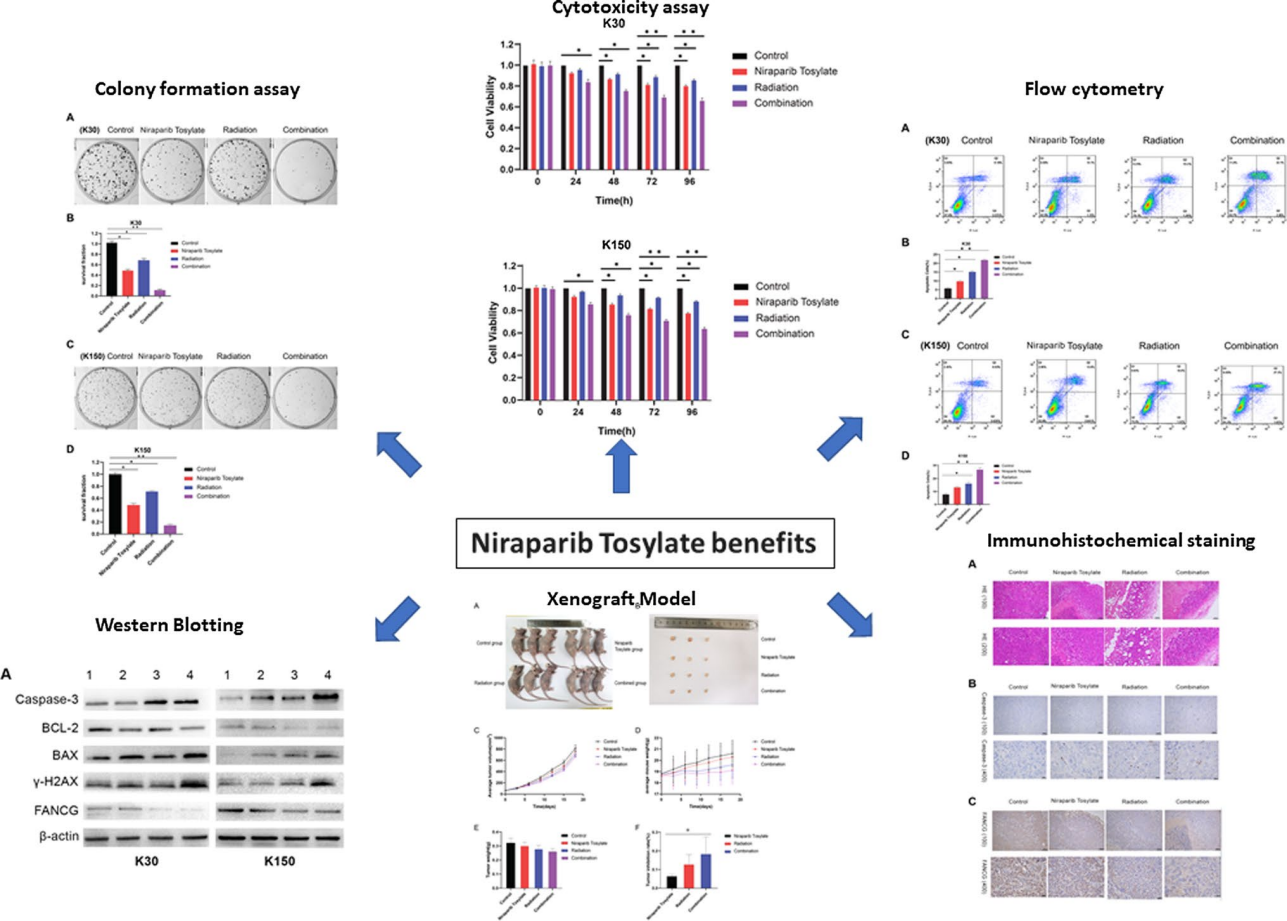
Diagrammatic representation to highlight findings of the study

After extracting cell protein and tumor tissue protein, Western blotting results showed that FANCG expression decreased in the niraparib tosylate group and the radiotherapy group, and the FANCG expression was the lowest in the combined treatment group, with statistically significant differences (*P* < 0.01) (Fig. 4-1 and Supplementary Fig. 5). A total of 28,567 genes were detected by transcriptome sequencing of transplanted tumor, and 349 genes were significantly different, including 316 up-regulated genes and 33 down-regulated genes. The difference in FANCG expression was statistically significant (*P* < 0.01). (Supplementary Fig. 4). Western blotting results showed that FANCG expression was decreased in the niraparib tosylate group and the radiotherapy group, and the FANCG expression was the lowest in the combined treatment group, with statistically significant differences (*P* < 0.01) (Fig. 4-1). After overexpression of FANCG, the inhibition of xenograft tumor was significantly reduced in both the radiotherapy group and the combined group, and there was no statistical difference in tumor weight between the four groups (*P* > 0.05). There was statistically significant difference in tumor inhibition rate between the combined group and the control group (*P* < 0.05), but there was no statistically significant difference in other groups (Fig. 6). As revealed from Kaplan–Meier plot, the differential expression of FANCG in esophageal squamous cell carcinoma cells showed no difference in survival (Fig. 7). In vitro and in vivo experiments at the molecular level demonstrated that niraparib tosylate can target FA-BRCA signalling and reduce FANCG expression, thereby enhancing radiosensitivity. A graphical description of the proposed pharmacological mechanism of niraparib tosylate drug is provided in Fig. 8 and findings for the current proposal are illustrated in Fig. 9.

## Discussion

In this study, based on the inhibition of cell proliferation and increased cytotoxicity in cell lines treated with radiotherapy and niraparib, we confirm that niraparib demonstrates radiosensitizing ability in EsC and could be used in clinical studies. Further, molecular testing after xenograft experiments revealed FANCG significant reduction in cells treated with combination therapy and hence, we speculate that the radiosensitivity effect of niraparib tosylate may depend on the regulation of FANCG expression. To verify this hypothesis, we first tested FANCG expression in KYSE-30 and KYSE-150 cells in vitro by Western blot. We found that the addition of niraparib tosylate significantly down-regulated FANCG protein expression compared with the control group. Therefore, the expression of FANCG in the FA-BRCA pathway showed a trend of steady decline after the combination of niraparib tosylate and radiotherapy. These results confirm our hypothesis that niraparib tosylate plays a radiosensitization role by down-regulating FANCG expression in the FA-BRCA pathway. To further verify this hypothesis, we used lentivirus to overexpress FANCG gene in KYSE-30 cells, and then carried out the transplantation experiment in tumor-bearing nude mice again.

Currently, radiotherapy is used for approximately 50% of all cancers including esophageal cancer. This has resulted in a cure rate of 40% [20]. Radiosensitivity is usually influenced by a number of factors, including tumor intrinsic radiosensitivity, tumor repair ability reoxidation processes, tumor tissue reaggregation and cell cycle redistribution. Ionizing radiation induces DNA damage, including SSB, base modification, cross-linking, and DSB. Failure of the DNA damage response (DDR) can lead to permanent cell cycle arrest or programmed cell death [21].

Niraparib tosylate is PARPi, a member of the PARP family, which binds to DNA SSB and DSB through its N-terminal zinc finger [22]. By binding to its C-terminal catalytic domain, it niraparib inhibits the activity of PARP and produces inner and branched polymers of ADP-ribose (PAR) chains that extend over hundreds of ADP-ribose units [23–25]. Studies have shown that PARP1 initiates and regulates multiple DNA repair pathways [26]. PARP1 may also bind to short single-chain and recruit Mre11 in homologous recombination (HR), thus playing a crucial role in maintaining genomic integrity [27]. There is evidence that the loss of PARP1 causes hypersensitivity to ionizing radiation [28].

The DSBs created by niraparib tosylate, are either repaired or remain unrepaired which leads to synthetic lethality [29]. An in vivo study, evaluating the preclinical pharmacokinetics and anti-tumor efficacy of ABT-888 in combination with temozolomide, platinum, cyclophosphamide, and ionizing radiation, demonstrated that ABT-888 is a formidable PARP inhibitor with good bioavailability [30]. Our study, showed similar results. Niraparib tosylate alone proved to be a weak inhibitor on tumor growth, while both radiotherapy and combination therapy could effectively inhibit tumor growth in the xenograft model.

Recent clinical studies, have shown that PARPi are used as radiosensitizers to treat different types of cancers have proven effective. Besides being a good radiosensitizer, niraparib enhanced the anti-tumor immune effects of radiation on EGFR-mutated non-small cell lung cancer as seen from clone formation and apoptosis assay [31]. A phase I/II study (NCT03644342) to determine the safety, tolerability and efficacy of niraparib with radiotherapy for the treatment of metastatic invasive carcinoma of the cervix is currently ongoing [32]. The combination of niraparib with standard combination radiation therapy and androgen deprivation therapy for treating high risk prostate cancer patients is being evaluated in another phase II clinical trial (NCT04037254) [33]. A phase I study (NCT01390571) assessing the combination of Olaparib with radiotherapy with and without temozolomide in patients with relapsed glioblastoma, demonstrated that the combination is safe and well tolerated [34]. A similar phase I study (ISRCTN52658296), assessing radiotherapy plus Olaparib for newly diagnosed glioblastoma also led to favourable results [34]. Further phase II evaluation is underway.

One of the main functions of the FA-BRCA pathway is to coordinate the repair of inter chain DNA inter strand cross-links (ICLs) [35]. ICLs have a direct physical blocking effect on DNA replication and RNA transcription, which, if not properly repaired, can lead to cytotoxicity and chromosomal structural aberrations [36]. These 22 FANC proteins, together with many FA-associated factors, recognize ICL damage and initiate downstream double-strand break (DSB) repair.

In our study, the addition of niraparib tosylate significantly down-regulated FANCG protein expression compared with the control group. A steady decline in the FANCG expression was observed. These results confirmed that niraparib tosylate plays a radiosensitization role by down-regulating FANCG expression in the FA-BRCA pathway.

The study is not without limitation. First, it is a preclinical study and hence the findings need to be confirmed in clinical studies. However, the findings of this research may lay a foundation for evaluating the application of PARP inhibitors in esophageal squamous cell carcinoma patients in the future.

## Conclusion

The extensive molecular analysis performed in the study may assist in concluding that niraparib combined with radiation can further inhibit the proliferation of esophageal squamous cell carcinoma cells, promote cell apoptosis, and have a significant inhibitory effect on tumors. Through further study of the mechanism, we found that niraparib tosylate has a radiosensitization effect on esophageal squamous cell carcinoma cells by down-regulating FANCG expression in FA-BRCA pathway.

## Supplementary Information

Below is the link to the electronic supplementary material.

Supplementary figure 1: The median inhibitory concentrations (IC50) of Niraparib Tosylate at 24 hours (TIF 8359 kb)

Supplementary figure 2: Colony formation assay of five cell lines. A. Formation of colonies in five cell lines at different radiation doses. B Survival fraction of five cell lines (TIF 6745 kb)

Supplementary figure 3 (A, B and C): Immunohistochemical staining of tumor tissues in four groups. A. HE staining for KE30 cell line B. Caspase-3 staining for KE30 cell line. C.FANCG immunochemical staining for KE30 cell line. (PNG 1648 kb)

Supplementary figure 4 (A and B): A. Results of high-throughput sequencing. B. The 20 genes with the most significant differences in expression (TIF 7196 kb)

Supplementary figure 5: Western blotting of Caspase-3 and FANCG after extracting tissue protein from mouse tumor (PNG 87 kb)

## Abbreviations

EsC: Esophageal cancer
SCC: Squamous cell carcinoma
EAC: Esophageal adenocarcinoma
GEJ: Gastroesophageal junction
ECF: Epirubicin, cisplatin, and fluorouracil
FA: Fanconi anemia
SSB: Single-strand break
DSB: Double-strand break
DDR: DNA damage response
PARPi: PARP inhibitor
ICL: Interstrand cross-links
PBS: Phosphate buffered saline
