# Correction to: Therapeutic benefits of niraparib tosylate as radio sensitizer in esophageal squamous cell carcinoma: an in vivo and in vitro preclinical study

**DOI:** 10.1007/s12094-022-03020-5

**Published:** 2022-12-06

**Authors:** Yuzhong Cui, Wei Huang, Feng Du, Xiaoyang Yin, Lei Feng, Baosheng Li

**Affiliations:** 1 Tianjin Medical University Cancer Institute and Hospital, Tianjin, 300060 China; 2 National Clinical Research Center for Cancer, Tianjin, 300060 China; 3 Key Laboratory of Cancer Prevention and Therapy, Tianjin, 300060 China; 4 Tianjin’s Clinical Research Center for Cancer, Tianjin, 300060 China; 5 Department of Oncology, Zibo Municipal Hospital, Zibo, 255400 China; 6 Shandong First Medical University and Shandong Academy of Medical Sciences, Jinan, 250117 China; 7 Department of Radiation Oncology, Shandong Cancer Hospital and Institute, Shandong First Medical University and Shandong Academy of Medical Sciences, 440 Jiyan Road, Huaiyin District, Jinan, 250117 Shandong China

**Correction to: Clinical and Translational Oncology (2022) 24:1643–1656** 10.1007/s12094-022-02818-7

Following publication of this article, errors were identified in Fig. 4; specifically:

- Fig. 4-2B: in the immunofluorescence of K150-BCL2-24H, the following images were originally duplicated in error:
  - Combination (second row)
  - Niraparib Tosylate (3rd row)
- Fig. 4-2C: in the immunofluorescence of K30-γH2AX(24H), the following image was originally duplicated in error:
  - Radiation (1st row)

The images have been replaced with the correct images from the raw data.

The corrections do not have any effect on the final conclusions of the paper. The original article has been corrected.
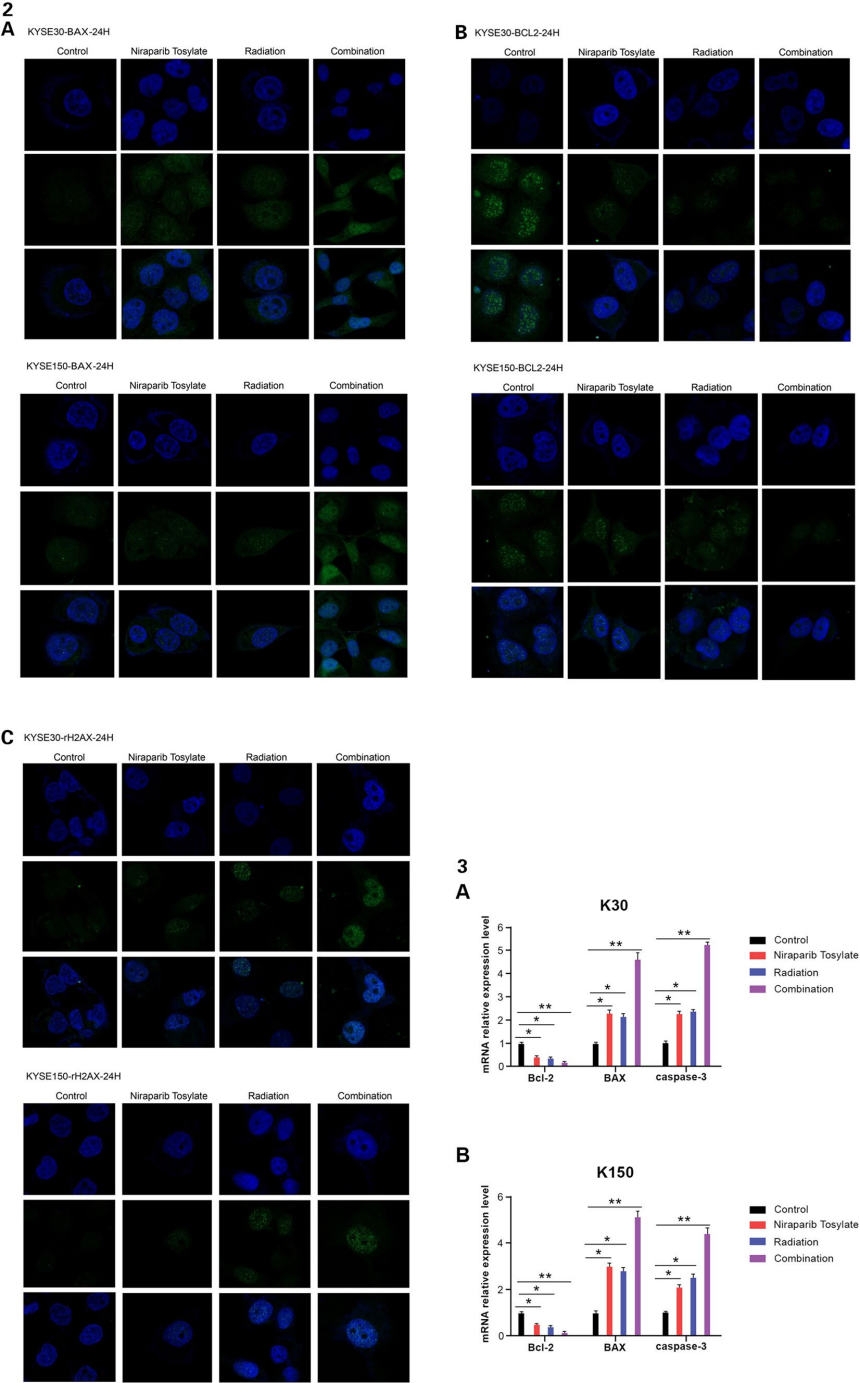


Figure 4 (2) **A** Immunofluorescence of γH2AX. **B** Immunofluorescence of BAX. **C** Immunofluorescence of BCL-2. (3) mRNA relative expression level among the four groups for **A** KYSE-30 cell line. **B** KYSE-150 cell line. *P < 0.05, **P < 0.01

