# Supplementary figures and images for: Therapeutic benefits of niraparib tosylate as radio sensitizer in esophageal squamous cell carcinoma: an in vivo and in vitro preclinical study
Source: Clin Transl Oncol. 2022 Apr 1;24(8):1643–56. doi: 10.1007/s12094-022-02818-7 (PMC9283188; doi:10.1007/s12094-022-02818-7)

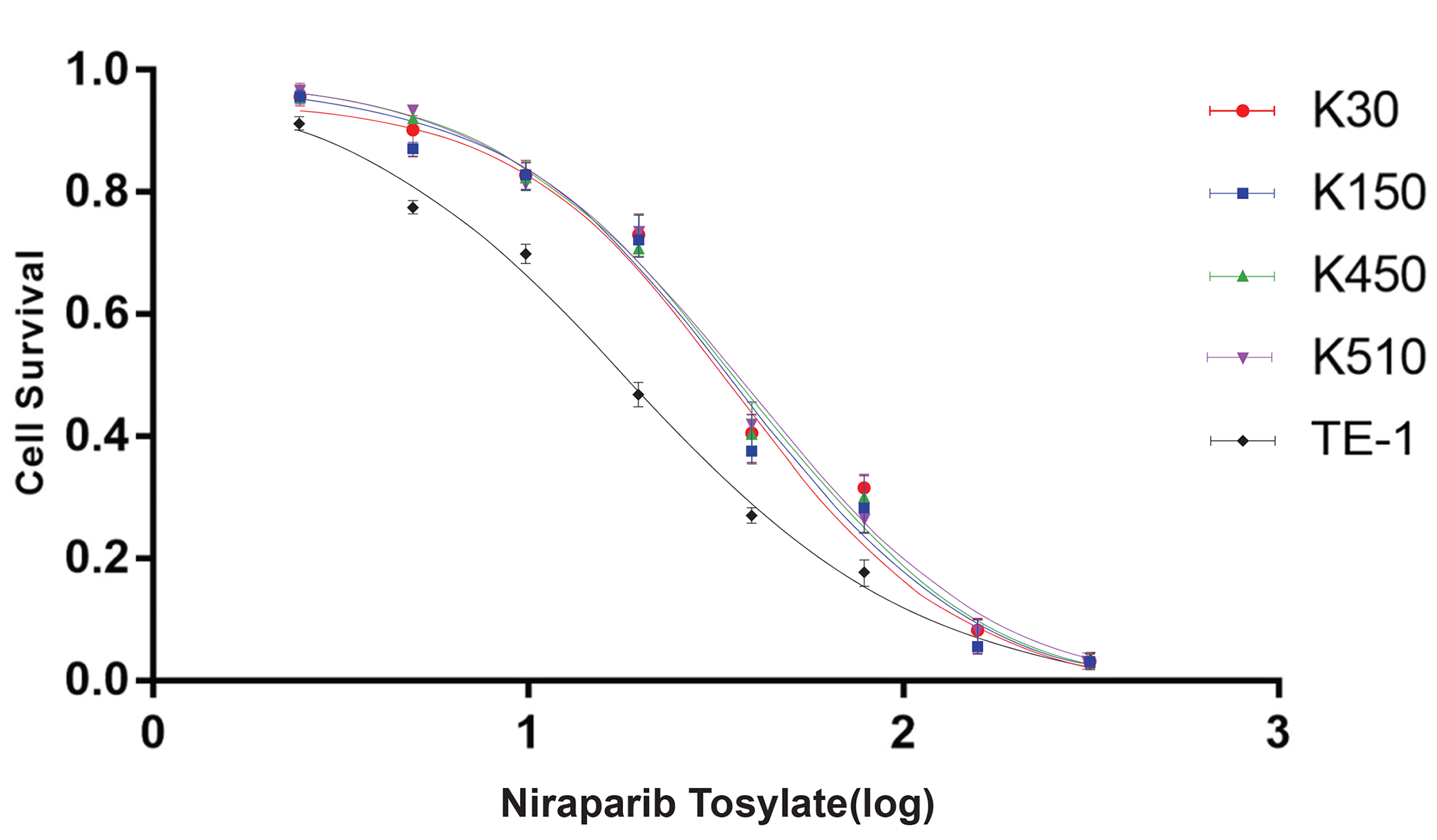

Supplement: Supplementary file 1 — Supplementary figure 1: The median inhibitory concentrations (IC50) of Niraparib Tosylate at 24 hours (TIF 8359 kb) [file 12094_2022_2818_MOESM1_ESM.tif]

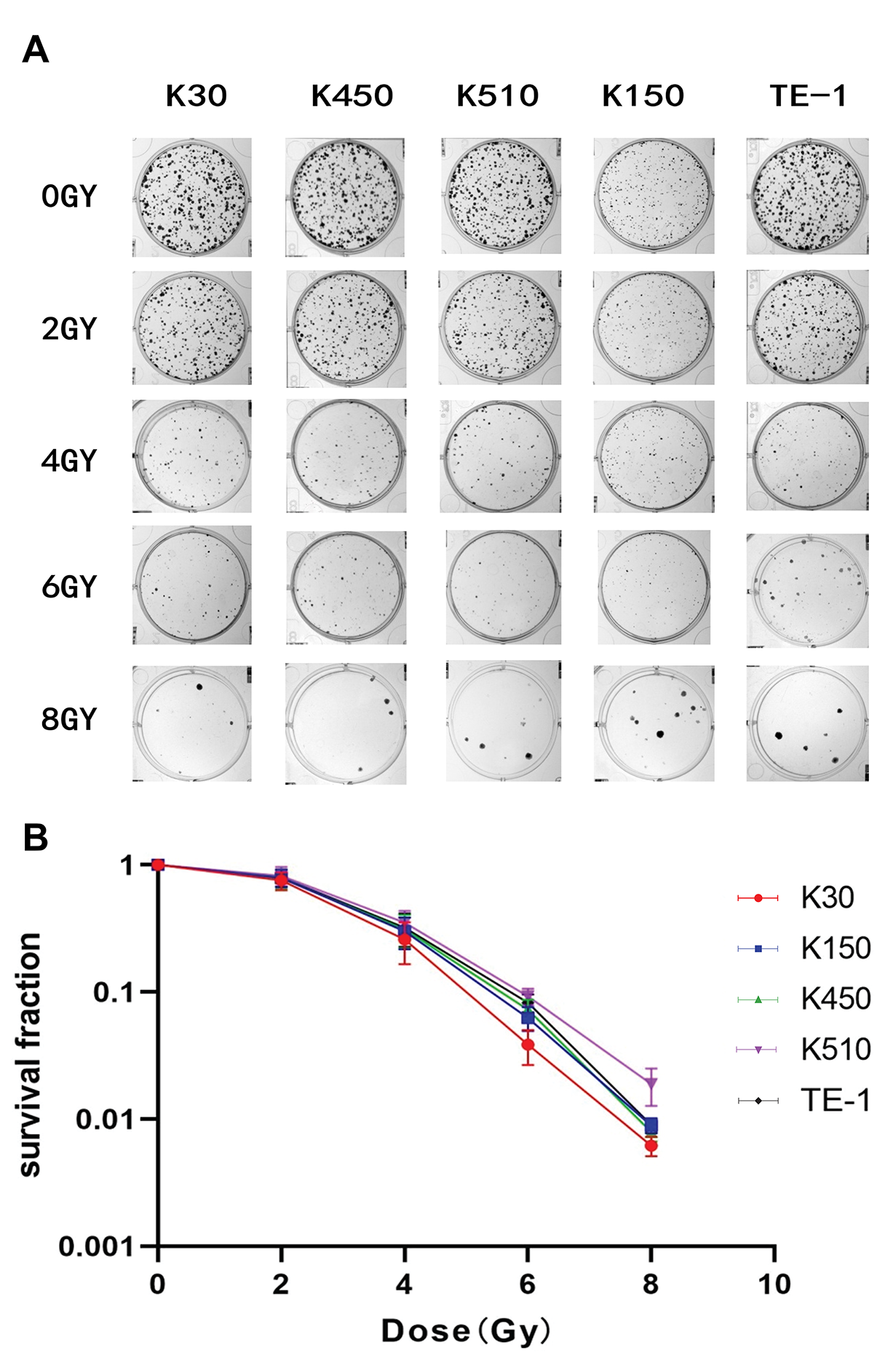

Supplement: Supplementary file 2 — Supplementary figure 2: Colony formation assay of five cell lines. A. Formation of colonies in five cell lines at different radiation doses. B Survival fraction of five cell lines (TIF 6745 kb) [file 12094_2022_2818_MOESM2_ESM.tif]

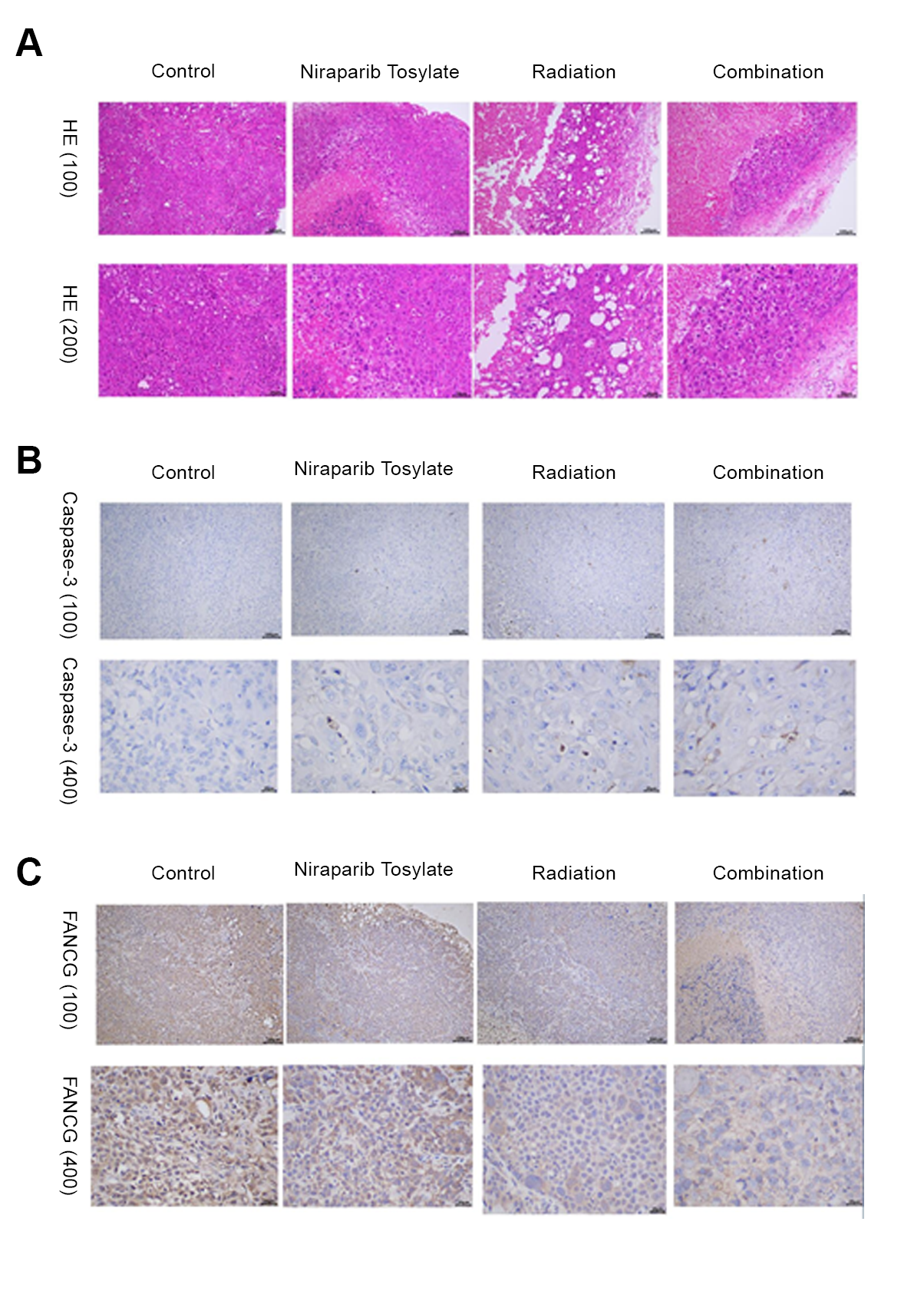

Supplement: Supplementary file 3 — Supplementary figure 3 (A, B and C): Immunohistochemical staining of tumor tissues in four groups. A. HE staining for KE30 cell line B. Caspase-3 staining for KE30 cell line. C.FANCG immunochemical staining for KE30 cell line. (PNG 1648 kb) [file 12094_2022_2818_MOESM3_ESM.png]

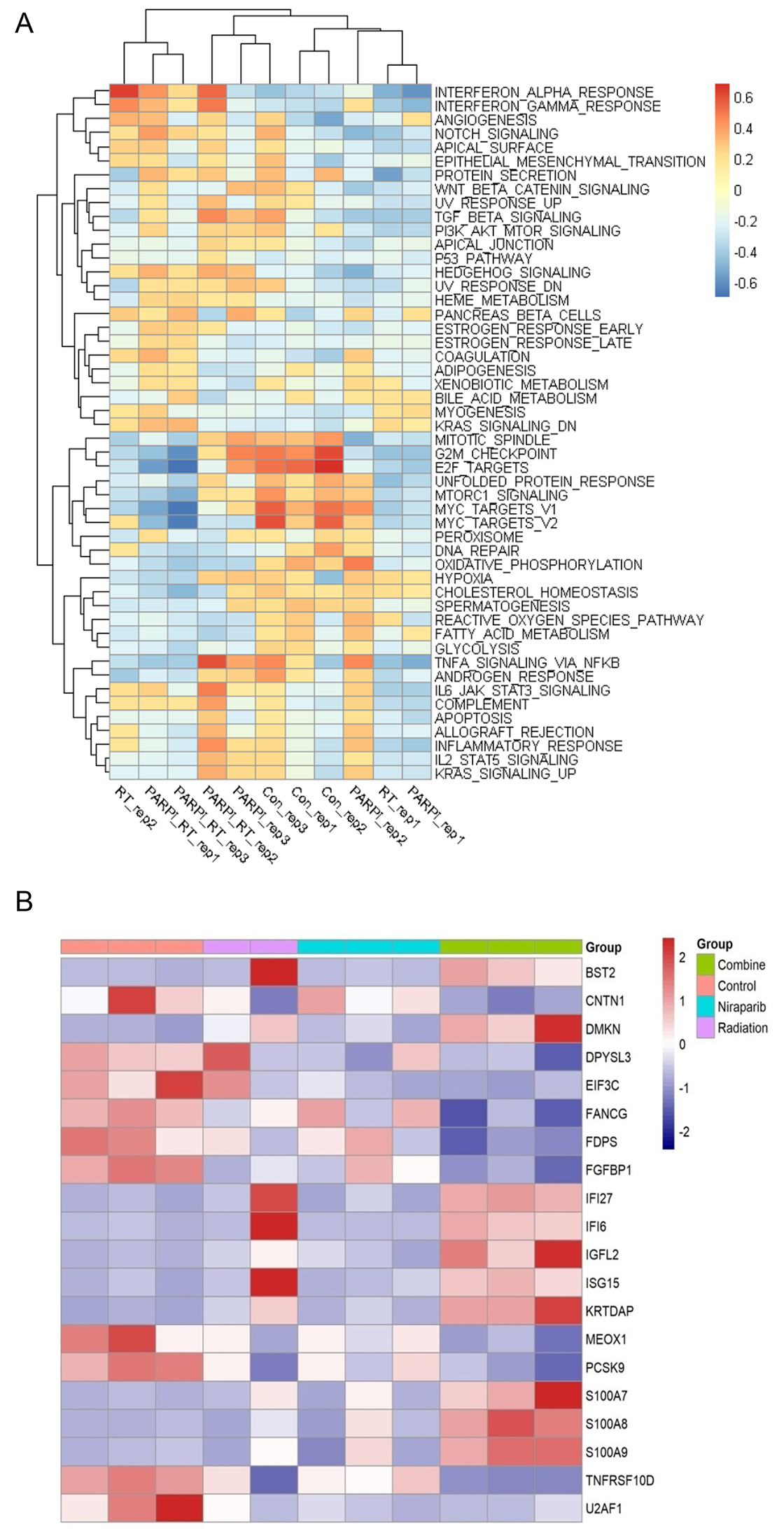

Supplement: Supplementary file 4 — Supplementary figure 4 (A and B): A. Results of high-throughput sequencing. B. The 20 genes with the most significant differences in expression (TIF 7196 kb) [file 12094_2022_2818_MOESM4_ESM.tif]

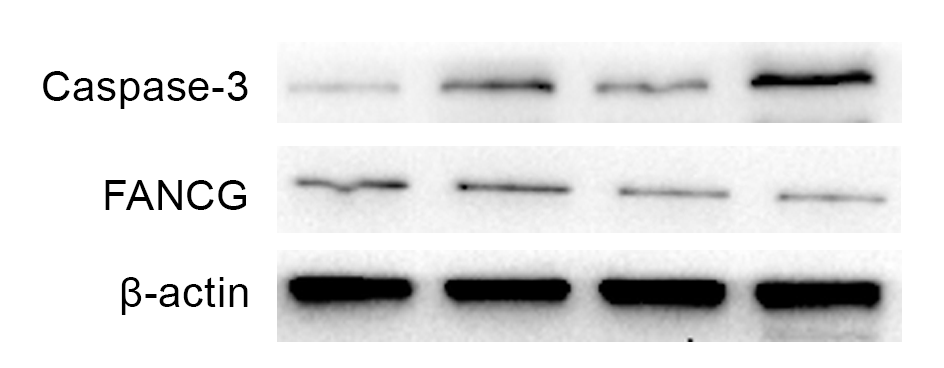

Supplement: Supplementary file 5 — Supplementary figure 5: Western blotting of Caspase-3 and FANCG after extracting tissue protein from mouse tumor (PNG 87 kb) [file 12094_2022_2818_MOESM5_ESM.png]
